# Barriers and Willingness to Undertake Cardiopulmonary Resuscitation Reported by Medical Students Dependent on Their Place of Residence—A Single-Center Study

**DOI:** 10.31083/j.rcm2512451

**Published:** 2024-12-23

**Authors:** Filip Jaskiewicz, Jakub R. Bieliński, Adam Jedrzejczak, Riley Huntley

**Affiliations:** ^1^Emergency Medicine and Disaster Medicine Department, Medical University of Lodz, 90-419 Lodz, Poland; ^2^School of Nursing, University of British Columbia, Vancouver, BC V6T 2B5, Canada

**Keywords:** out-of-hospital cardiac arrest, cardiopulmonary resuscitation, CPR, resuscitation, education

## Abstract

**Background:**

Bystander-administered cardiopulmonary resuscitation (CPR) is crucial for the survival of out-of-hospital cardiac arrests. However, only roughly 58% of bystanders would provide CPR, with wide variations across different regions. Identifying each factor affecting the barrier or readiness to perform resuscitation is a significant challenge for researchers. This study aimed to evaluate the obstacles preventing first-year medical students from initiating CPR, focusing on the size of domestic residential environments and the time and methodology of practical training.

**Methods:**

The original online questionnaire surveyed first-year medical students at the Medical University of Łódź from February 1 to March 2, 2024. The questionnaire development involved a literature review, expert evaluation, and pilot testing. Participation was voluntary and anonymous, with strict inclusion and exclusion criteria. The data were analyzed using PQStat software, employing descriptive statistics.

**Results:**

The study revealed that 271 medical students reported a similar median of barriers regardless of the place of residence (median (Me) = 5, interquartile range (IQR) 2–6.25 vs. Me = 4, IQR 3–6 vs. Me = 4, IQR 3–6, *p *= 0.620). Out of 18 analyzed barriers, the only significant difference was found for crowded places. Medical students living in cities most frequently reported a willingness to perform CPR with rescue breaths for all victims. Those who grew up in towns <100,000 residents were less willing to start CPR if an unknown adult were the victim (rural area: 39.2% vs. town: 17.6% vs. city: 45.1%, *p* < 0.01). The number of reported barriers was similar regardless of training type and the time since training; however, the nature of these barriers varied after a year passed.

**Conclusions:**

Respondents across various locations reported similar number and types of barriers to performing CPR, including the most commonly declared fear of harm, uncertainty about recognizing cardiac arrest, and concerns about disease transmission. Although differences connected to the type of victims were observed, its low or moderate practical significance needs more comprehensive research on the impact of the size of the place of residence on the willingness to perform resuscitation and the related barriers.

## 1. Introduction

Out-of-hospital cardiac arrest (OHCA) is a public health problem worldwide [1, 2, 3]. Global epidemiological data suggest an average annual incidence of approximately 55 adult cases per 100,000 population, with rates as high as 170 cases per 100,000 in Europe [1, 4]. Only in 2015 in the United States were there 347,322 emergency medical service-assessed cases of adult OHCA, constituting approximately 1000 people each day, with a high mortality rate of up to 90% [5]. Cardiopulmonary resuscitation (CPR) administered by a bystander is the sole intervention mechanism capable of sustaining and ultimately restoring the life functions of victims with OHCA, occupying a critical link within the “Chain of Survival” [3, 4, 5, 6]. Findings from the European Registry of Cardiac Arrest (EuReCa) TWO project, created by the European Resuscitation Council (ERC) as an international multi-center survey of epidemiology, treatment, and outcome of victims suffering from OHCA in Europe, indicate that only half of bystanders provide CPR [2]. The study, conducted across 27 European countries, revealed substantial variations in the rates of bystander-administered CPR. These ranged from as low as 1 out of 10 individuals in Serbia to as high as 8 out of 10 in Norway. Such large discrepancies in the frequency of provided CPR are influenced by a wide range of factors, some of which have not yet been fully identified.

Scientific literature suggests the existence of external stimuli that can trigger a bystander’s response in the form of stress, hysteria, or panic [7, 8, 9, 10]. These stimuli, including the victim’s state of health, general appearance, age, gender, and perceived chances of survival, may discourage bystanders and cause psychological barriers, reducing their willingness to provide basic life support (BLS) [9, 10, 11, 12, 13]. OHCA victims with vomit, unpleasant odors, or visible blood often cause bystanders to feel disgusted or afraid of contracting diseases [7, 8, 9, 10, 11, 12]. Other factors can cause fear of harming the victim or facing legal consequences [11, 12, 13, 14, 15, 16]. Furthermore, the bystander’s perception of their competence, knowledge, or physical strength can undermine their self-confidence and lower their ability to perform CPR [11, 12, 13, 14, 15, 16, 17]. Evidence demonstrates that numerous external stimuli have been studied, and their impact on inducing psychological triggers in the general public has been determined. However, it is less common to strictly separate individuals and groups beyond the public and analyze which specific populations or subgroups are distinct from the broader community regarding their responses and behaviors.

Current evidence indicates that the willingness to learn or the confidence to perform bystander CPR is affected by the socioeconomic status and origins of the first aid rescuer. Members of low-income urban populations frequently show little interest in BLS training unless the training closely relates to their work duties [7]. Bystander CPR is typically lower in rural areas and in poorer neighborhoods. The chances of surviving OHCA are reduced in low-income communities due to fewer chances of receiving help from witnesses [18]. In comparison, residents from the least socially deprived areas are significantly more likely to receive bystander CPR than those in socioeconomically marginalized communities [19].

However, limited research has been conducted on analyzing specific differences in psychological motivation for undertaking CPR between rural–urban populations. Researchers have gathered evidence showing different chances of surviving the OHCA depending on where it occurred; however, more focus is required on the causes that are most likely related to the psychological barriers stopping witnesses from performing CPR. Demographic and community factors logically influence individual traits, which may affect the occurrence of psychological barriers. Variations in the size of the provider’s residential environment or the time since BLS or the first aid course in which they participated can significantly shape their attributes and perspectives. In addition, since it has been proven that there is a difference in the interest in BLS training depending on the size of the place of origin, it would be necessary to assess the presence of practical courses and their quality among these groups [7]. Disparities across small rural villages, medium-sized urban towns, and large metropolitan cities, such as accessibility to medical resources, frequency of emergency response training, and cultural attitudes towards medical crises, may result in distinct bystander attitudes.

The first-year medical student cohort is a critically important population requiring further investigation and will likely provide data from various administrative divisions. As aspiring healthcare providers, these novice practitioners ought to possess fundamental BLS knowledge recently, and their preparedness to perform CPR as bystanders is of paramount importance, as corroborated by the standards set forth by the British General Medical Council [20, 21, 22].

“You must offer help in an emergency, taking account of your own safety, your competence, and the availability of other options for care.” [23].

Many barriers have been studied, but the rural–urban differences among medical students, with an additional analysis of their last CPR training, need further research. Investigating specific cohorts, analyzing their differences, and incorporating these findings to tailor educational systems worldwide could strengthen global healthcare quality. Continued study and implementation of evidence-based strategies are highly important to promote a culture of emergency preparedness and help individuals act when facing OHCA. It is essential to ensure that future healthcare professionals, regardless of origin and regional background, are well-equipped and self-assured in performing bystander CPR for OHCA victims.

### Aim

The general aim of this study was to evaluate the barriers preventing first-year medical students from initiating CPR and compare these data with the size of their domestic residential environments and the time and methodology of their last practical training. For detailed purposes, the following were analyzed:

• The relationship between the approximate size of the administrative division where the respondents lived (rural area, e.g., countryside villages, town under 100,000 inhabitants, city over 100,000 inhabitants).

• The relationship between the exact population size in the respondents’ residence and the occurrence of barriers to resuscitation.

Furthermore, to provide clarity and a comprehensive perspective, this study examines the association between the timing and methodology of practical training and the most prevalent barriers.

## 2. Materials and Methods

### 2.1 Study Group and Study Procedure

This cross-sectional study was conducted using an original survey questionnaire. Data were collected from February 1, 2024, to March 2, 2024, among first-year medical students at the Medical University of Łódź. Information about the possibility of participating in the study, along with a link to the questionnaire, was sent to respondents’ email addresses four times (on the day the study began and 12, 6, and 2 days before the planned end of the study). The survey software allowed respondents to submit their responses only once. The study received approval from the Bioethics Committee of the Medical University of Lodz (No. RNN/41/24/KE). Informed consent was obtained from all participants.

Participation in the study was voluntary and anonymous. The inclusion criteria were first-year medical student status, willingness to participate in the study, and the return of a fully completed questionnaire. The exclusion criteria included lack of consent to participate in the study, completion of other medical education or practicing another medical profession, and failure to answer any question.

### 2.2 Data Sources/Measurements

The sample size of the study was calculated using G*Power version 3.1.9.7 (Heinrich-Heine-Universität Düsseldorf, Düsseldorf, Germany). Based on the number of all first-year medical students from the 2023/2024 intake at the Medical University of Łódź (*N* = 533), at least 223 respondents were needed to achieve a confidence level of α = 0.95 and a maximum error of 0.05 (population proportion of 50%). The survey questionnaire was generated using Microsoft Forms software (ver. 18.2306.1061.0, Microsoft Corporation, Redmond, Washington, DC, USA). The questionnaire form consisted of 17 questions: four concerning the basic characteristics of participants, six focused on the CPR training history, three connected to types of victims in which respondents would start the CPR, two about declared barriers (one with closed answers and one open-ended question: to allow for more nuanced, honest and clear explanations, then categorized) and two questions about the size of the place of residents (one closed answer in categories: rural, town <100,000 habitants, city >100,000 habitants and one open-ended to provide exact number of residents: an online source for easy and unified verification was proposed) (**Supplementary Material 1**). Moreover, a complete confidentiality assurance to encourage honest responses was strongly articulated in emails containing the survey to minimize social bias in your questionnaire.

The process of creating and evaluating the questionnaire was as follows:

Stage 1: A literature review to identify questions used in studies on similar topics, including literature reviews, original articles, guidelines, and international recommendations.

Stage 2: Analysis of questions contained in other researchers’ questionnaires and their critical verification for consistency with the aims of the presented study.

Stage 3: Preparation of an appropriate list of questions verifying the study’s objectives.

Stage 4: Substantive evaluation by three subject matter experts.

Stage 5: Presentation of the pilot survey to 31 respondents to assess whether it:

• is understandable to respondents

• is interpreted similarly by all respondents

• suggests any bias on the part of the researcher.

Stage 6: Finalization of the questionnaire evaluation: Due to its simplicity and short form, the more detailed validation of the research tool (e.g*.*, Cronbach’s alpha test) was abandoned or irrelevant to the evaluated questionnaire.

### 2.3 Statistical Analysis

Statistical analyses were performed using PQStat 1.8.4.152 software (PQStat Software, Poznań, Poland). Quantitative variables are presented using basic descriptive statistics: arithmetic mean (x), standard deviation (SD), median (Me), interquartile range (IQR), and percentages (%). The Kruskal–Wallis test was used to compare the number of reported barriers of respondents by the three groups (rural, town, and city). The post-hoc Dunn’s comparison test was performed with Bonferroni correction to compare the different groups. The Chi-square test and Fisher’s exact test were used to compare the distribution of responses between the three groups to identify the type of victims for whom respondents would perform resuscitation. Spearman’s rank correlation coefficients were estimated to analyze responses by the size of the place of residence. A test probability of *p* < 0.05 was considered significant, and a test probability of *p* < 0.01 was considered highly significant.

## 3. Results

A total of 273 out of 533 first-year medical students at the Medical University of Łódź participated in the study. In total, 271 completed questionnaires met the inclusion criteria (2 were rejected due to incomplete data). The average age of the respondents was 19.5 ± 1.6 years (Me = 19; min = 18; max = 33). Females constituted 65.6%, and 2.2% of respondents refused to provide their gender. Further characteristics of the study group are presented in Table 1.

**Table 1.** **Respondent characteristics in terms of place of residence size and past training**.

Characteristics (*N* = 271)		Study group	*n*	%
Place of residence		Rural area (countryside villages)	84	30.1
		Town (<100,000 inhabitants)	85	31.4
		City (>100,000 inhabitants)	102	37.6
Prior first aid training		Yes, <1 year	111	41
		Yes, >1 year	160	59
		No	0	0
Types of prior practical (hands on) first aid training				
	Assess consciousness and breathing	Yes	221	81.5
		No	50	18.5
	CPR on an adult manikin	Yes	245	90.4
		No	26	9.6
	CPR on a child manikin	Yes	105	38.7
		No	166	61.3
	AED use	Yes	91	33.6
		No	180	66.4

*N*, population size; *n*, sample size; CPR, cardiopulmonary resuscitation; AED, automated external defibrillator.

The study showed that the number of barriers experienced by individual respondents ranged from 1 to 16, with the majority reporting between 3 and 6 barriers (Table 2). This result was consistent across all three groups (ranging from 2 to 6 in rural areas), indicating that the number of barriers in the studied group is not dependent on the population size of the home region.

**Table 2.** **The frequency of barriers reported by respondents depends on their place of residence**.

Place of residence		Me	IQR	*n*
Rural area		5	2–6.25	85
Town		4	3–6	85
City		4	3–6	102
*p*-value		0.620		
	*η2 = 0.0039*			

Me, median; IQR, interquartile range; *n*, sample size; η2, Eta-squared.

The most frequently reported barriers were related to legal consequences, fear of contracting disease from the victim, fear of causing harm, lack of knowledge, lack of confidence, and uncertainty about correctly identifying cardiac arrest.

Only in the case of one response was a significant (χ^2^ = 6.06, *p *= 0.0482) difference observed between groups regarding the place of residence. This concerned cardiac arrest occurring in a place with many witnesses. This response was least frequently indicated by people from towns with under 100,000 inhabitants. In comparison, others (people from rural areas and cities with over 100,000 inhabitants) reported this barrier at a similar level (about 5 times more frequently, although the effect has low significance; Table 3). Additionally, a multivariate analysis of logistic regression predictions of each barrier with the factors: place of residence, gender, age and time of course completion was performed, which showed that none of the barriers was significantly related to place of residence (**Supplementary Material 2**).

**Table 3.** **The barriers and fears to initiating resuscitation reported by respondents depends on their place of residence**.

Declared barriers	Rural area		Town		City		χ ^2^	*φc*	*p*-value
	*n*	%	*n*	%	*n*	%			
Without a specific reason	0	0	2	2.3	4	3.9	3.28	0.11	0.193
Fear of legal consequences	33	39.2	28	32.9	26	25.4	4.06	0.12	0.131
Fear of contracting disease from the victim	43	51.1	38	44.7	52	50.9	0.94	0.05	0.622
Fear of contracting coronavirus	6	7.1	6	7.0	4	3.9	1.15	0.06	0.560
Fear of vomit	24	40.4	40	47.0	46	45.1	0.78	0.05	0.674
Fear of not having enough physical strength	18	21.4	14	16.4	16	15.6	1.17	0.06	0.556
Fear of a victim under the influence of alcohol	22	26.1	18	21.1	22	21.5	0.76	0.05	0.683
Fear of a bloodied victim	12	14.2	10	11.7	16	15.6	0.59	0.04	0.741
Fear of panicking	24	28.5	20	23.5	28	27.4	0.61	0.04	0.735
Fear of causing harm to the victim	43	51.1	41	48.24	56	54.9	0.83	0.05	0.658
Lack of self-confidence	36	42.8	25	29.4	30	29.4	4.69	0.13	0.095
Lack of adequate knowledge	30	35.7	27	31.7	28	27.4	1.47	0.07	0.479
Uncertainty if cardiac arrest was correctly identified	28	33.3	27	31.7	38	37.2	1.71	0.07	0.714
Low socio-economic status of the victim	37	44.0	46	54.1	50	49.0	2.47	0.09	0.424
Advanced age of the victim	12	14.2	12	7.8	8	14.1	0.09	0.01	0.290
The victim is a child	4	4.7	4	4.7	4	3.9	0.36	0.03	0.951
The victim is a woman	8	9.5	8	9.4	12	117	4.47	0.12	0.834
There are many witnesses at the scene	9	10.7	2	2.3	12	11.6	6.06	0.14	0.048

*n*, sample size; χ^2^, chi-squared; φc, Cramér’s V.

The questionnaire also assessed the relationship between the size of the place of residence and the type of victims for whom respondents would perform resuscitation. Two action options consistent with ERC and American Heart Association guidelines were analyzed: performing chest compressions only (Table 4) or the full algorithm of alternating compressions with rescue breaths (Table 5).

**Table 4.** **The willingness of respondents to initiate compression-only CPR depends on the victim type and place of residence**.

Type of victim	Rural area		Town		City		*φc*	*p*-value
	*n*	%	*n*	%	*n*	%		
Adult—family member	68	80.9	55	64.7	78	76.4	0.15	0.045
Adult—stranger	80	95.2	81	95.2	92	90.2	0.09	0.267
Child—family member or known to you	65	77.3	49	57.6	74	72.5	0.17	0.014
Child—stranger	71	84.5	67	78.8	86	84.3	0.06	0.529
Will not perform CPR on any victim	2	2.3	6	7.0	2	1.9	0.12	0.136

*n*, sample size; φc, Cramér’s V; CPR, cardiopulmonary resuscitation.

**Table 5.** **The willingness of respondents to initiate CPR depends on the victim type and place of residence**.

Type of victim	Rural area		Town		City		*φc*	*p*-value
	*n*	%	*n*	%	*n*	%		
Adult—family member	76	90.4	81	95.2	98	96.0	0.10	0.232
Adult—stranger	33	39.2	15	17.6	46	45.1	0.24	<0.001
Child—family member or known to you	68	82.1	69	81.1	90	88.2	0.08	0.351
Child—stranger	37	44.0	27	31.7	50	49.0	0.14	0.053
Any victim	8	9.5	6	7.0	2	1.9	0.13	0.080

*n*, sample size; φc, Cramér’s V; CPR, cardiopulmonary resuscitation.

The question the relationship between the size of the place of residence and the type of victims for whom respondents would perform resuscitation. Two action options consistent with ERC and American Heart Association guidelines were analyzed: performing chest compressions only (Table 3) or the full algorithm of alternating compressions with rescue breaths (Table 4).

Regarding the chest compressions-only version, a significant (*p *= 0.045) relationship was found between the frequency of indicating this type of victim and the respondents’ place of residence for adult family members. This response was least frequently indicated by people from towns with under 100,000 inhabitants and significantly more often by people from rural areas and cities with over 100,000 inhabitants.

A similar relationship was found for children from the family or immediate surroundings. A significant (*p *= 0.014) relationship was observed between the frequency of selecting this response and the place of residence; however, similar to adult family members, the effect was low (Table 4). As in the previous point, this response was least frequently indicated by people from cities with under 100,000 inhabitants and significantly more often by people from rural areas and cities with over 100,000 inhabitants.

No significant differences were observed for the other options. Therefore, it can be concluded that the place of residence does not matter in these cases (Table 4).

For the option with the algorithm including rescue breaths, data analysis showed a significant (*p* < 0.001) relationship between the place of residence and the respondents’ indication of readiness to perform CPR on an unfamiliar adult, and this time with moderate effect. More frequently, residents of rural areas and cities indicated this response with over 100,000 inhabitants. People from towns with under 100,000 inhabitants chose this option significantly less often.

It should also be noted that people from cities with over 100,000 inhabitants most frequently reported willingness to perform CPR using chest compressions and rescue breaths for all types of victims (Table 5).

Regarding the other responses, no significant relationship was observed between the place of residence and selection. No significant correlations were found between the number of responses to the questions about barriers/fear of starting resuscitation and the size of the place of residence. This includes questions on performing only chest compressions or both chest compressions and rescue breaths.

**Fig. 1.**
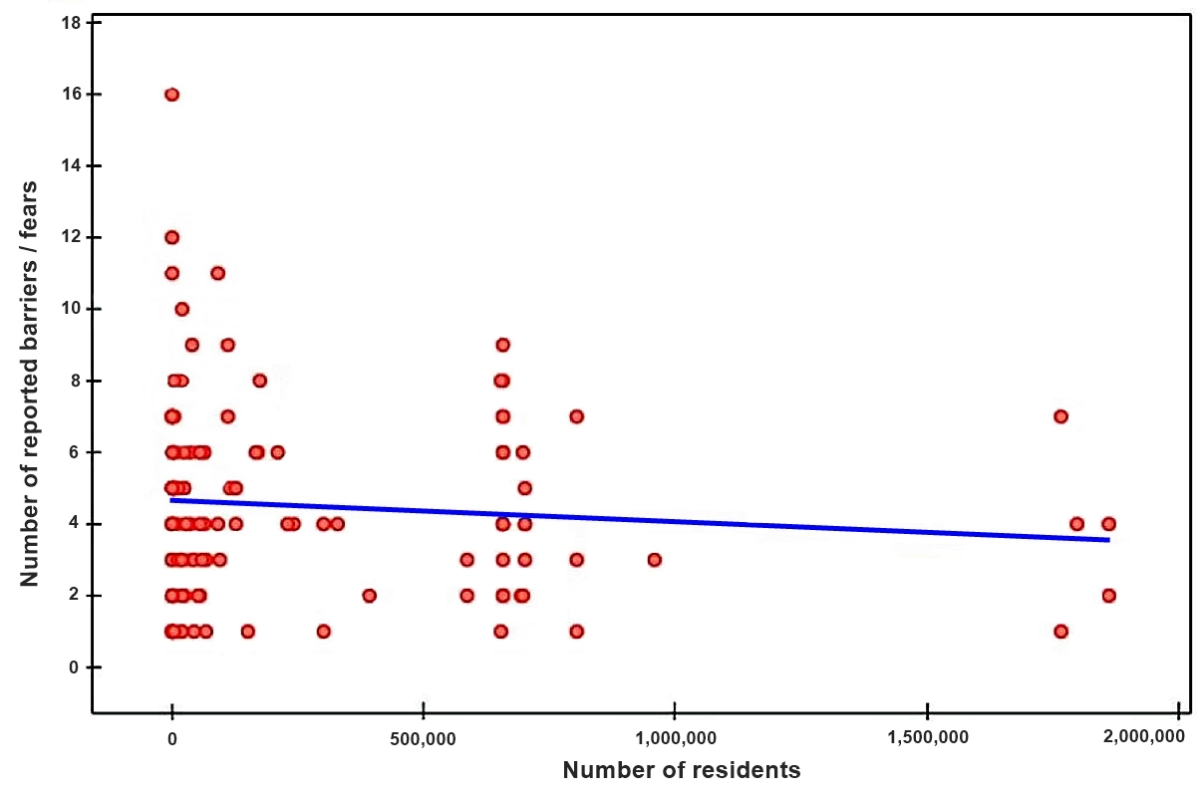
**Distribution of the number of barriers to initiating resuscitation reported by respondents depending on the exact reported number of residents in the respondent’s place of residence**.

The detailed number of barriers among people from places of different population sizes was also analyzed using Spearman’s rank correlation, R = –0.0852, *p *= 0.1619. The study suggests no significant correlations exist between the number of responses to the question “Select all statements that define your barriers/fears about starting resuscitation” and the place of residence concerning the exact population size provided by respondents. Therefore, it can be concluded that the place of residence does not affect the number of barriers preventing respondents from performing CPR (Fig. 1).

**Fig. 2.**
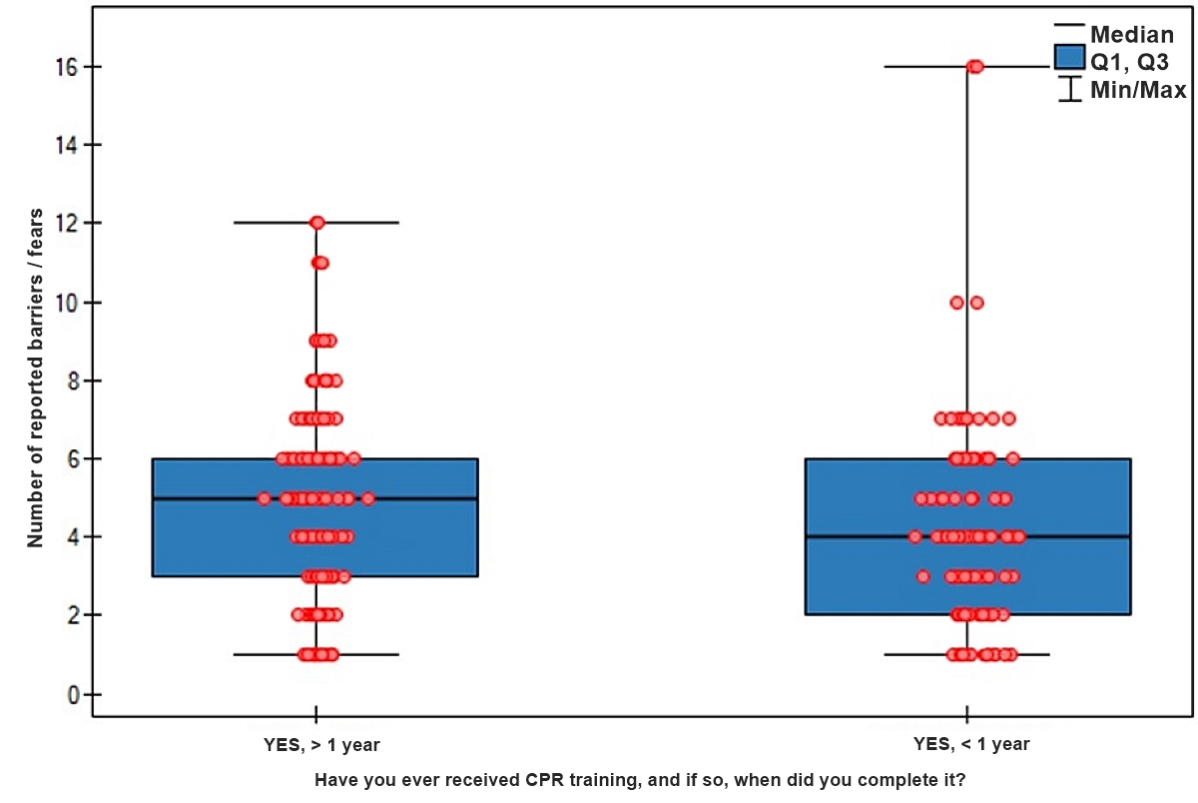
**Distribution of the number of barriers and fears to initiating resuscitation reported by respondents based on time elapsed since prior first aid training**. CPR, cardiopulmonary resuscitation.

Additionally, to offer a clear and comprehensive perspective, the study investigated the relationship between the timing and approach of practical training and the most common barriers encountered (Fig. 2).

The results indicate that the frequency of reported barriers did not vary significantly based on the time elapsed since the resuscitation training was completed (<1 year: Me = 4 (IQR 2–6) vs. >1 year: Me = 5 (IQR 3–6); *p* = 0.058). Nevertheless, the specific types of reported barriers demonstrate substantial variation depending on this time factor. Particularly, the barrier described as “Without a specific reason” is exclusively reported by those who had completed the course more than a year prior (χ^2^ = 4.32, *p* = 0.037). The “low socio-economic status of the victim” barrier is more frequently reported by the group that completed the course more than a year prior compared to those who had finished the training within the previous year (χ^2^ = 7.62, *p* = 0.005).

Moreover, concerns regarding contracting an illness (χ^2^ = 1.89, *p* = 0.169) or managing vomit (χ^2^ = 0.64, *p *= 0.421) did not exhibit significant differences based on whether respondents practiced both chest compressions and rescue breaths or solely compressions during the training. This study also analyzed the relationship between the number of barriers reported by the respondents and their previous training methodology and found no significant differences (R = –0.0621, *p* = 0.161). Likewise, the concern about accurately identifying cardiac arrest did not significantly differ depending on whether respondents practiced assessing consciousness and breathing (χ^2^ = 0.09, *p *= 0.762).

## 4. Discussion

The barriers identified in this study are among the most common that can impact respondents’ willingness to perform CPR, as reported by other researchers [7, 8, 9, 11, 12, 13, 14, 15, 16, 17, 24]. The most frequently reported barriers among all study respondents were:

• concern about potentially harming the victim (51.7%)

• fear of contracting an illness from the victim (49.1%)

• uncertainty regarding the accurate identification of cardiac arrest (34.3%)

• lack of self-confidence (33.6%).

The first three barriers are directly related to the provider’s proficiency in CPR and their knowledge of anatomy and physiology; it appears that they can be overcome with a well-conducted BLS course, as completing BLS training improves a provider’s competence and self-assurance in providing first aid [25, 26, 27]. However, the final barrier requires appropriate knowledge and adequate protection against disease-transmitting microorganisms during CPR. This issue could potentially be addressed by providing resuscitation masks. Furthermore, teaching rescuers that CPR primarily consists of chest compressions, while the effectiveness of rescue breaths remains undetermined according to the evidence, and reminding the provider that mouth-to-mouth ventilation is not mandatory could potentially mitigate the barrier associated with the fear of infectious disease transmission, thereby increasing the likelihood of bystander intervention in OHCA [28, 29, 30].

The study group was more inclined to perform CPR using only chest compressions on unfamiliar individuals. This concern applies to both adult and pediatric victims. As the literature suggests, this may be attributable to a fear of contracting illnesses from the victim [7, 8, 12]. Conversely, most respondents were willing to provide mouth-to-mouth ventilation solely to family members. This is understandable, as medical history is typically known in such cases, and the risk of infection is clear. Research by Fratta* et al*. [31] indicates that individuals display a markedly greater willingness to administer CPR when a family member’s life is at risk compared to when assisting a stranger. Just 44.8% of respondents would administer CPR on a stranger, compared to 68.6% that would do so on a family member. Notably, respondents from cities with populations exceeding 100,000 were more willing to perform comprehensive CPR on any type of victim, but respondents from rural areas were more likely to perform CPR without mouth-to-mouth ventilation in most situations (φ*c* = 0.24, *p* < 0.001).

One noteworthy response showed a significant difference between groups regarding the place of residence. This concerned OHCA occurring in a crowded location. Respondents from towns with fewer than 100,000 inhabitants were least likely to report this as a psychological barrier, while others noted it at a similar level, approximately five times more frequently. Still, according to a low number of responses and low effect (φ*c* = 0.14), this finding may not have a practical significance. This difference is also not currently explainable using evidence-based scientific knowledge, as it has not previously been observed. Bylow* et al*. [32] demonstrated that victims with OHCA occurring in crowded public places have the highest probability of survival, compared to other locations outside the hospital, due to the increased likelihood of prompt bystander intervention. However, the size of the administrative division where the research was carried out and the origins of the respondents were not discussed in the article. This requires more research since it might have important public health ramifications for training programs in basic life support, smaller communities, and bystander response in densely populated places.

The systematic review by Uny *et al*. [7] revealed the significant and specific barriers individuals in deprived communities face in becoming prepared to perform bystander CPR. Although individuals exhibited a strong willingness to intervene, they faced important barriers, including limited access to training programs and inadequate knowledge and skills in this domain. The findings reported by Uny *et al*. [7] appear to diverge from the experiences of medical students from rural and small-town areas. This discrepancy may be attributable to disparities in the availability of training and educational resources across different national contexts, with Poland potentially exhibiting a different landscape than the nations encompassed within the systematic review.

Interestingly, the factor of time elapsed since resuscitation training affects the specific types of reported barriers rather than the frequency of reported barriers. Jaskiewicz *et al*. [24] reported a similar trend; however, they identified different kinds of barriers, such as the fact that the victim was a child, fear of harming the child, fear of legal consequences, and insufficient knowledge of CPR.

In this study, the results show how the low social status of OHCA victims significantly affects bystanders’ willingness to assist. This effect was particularly notable among novice rescuers, specifically those who completed their BLS training more than a year earlier. Continuous training and reinforcement of resuscitation skills may be vital to address the changing barriers that bystanders face when responding to medical emergencies, not only for pediatric populations but also for individuals with low social status. Frequent refresher courses and simulation-based training, in line with recent International Liaison Committee on Resuscitation (ILCOR) recommendations, could help maintain the confidence and competence of potential rescuers, even as time passes since their initial certification [33]. Studies by Scapigliati *et al*. [34] and González-Salvado *et al*. [35] align with the view that repeated training and recertification of BLS and CPR courses can reduce barriers to performing CPR among the general public.

This ongoing project seeks to find and exclude potential factors affecting barriers to bystander CPR. However, it is necessary to note the complexity of this topic and its differences from many other research problems. In reference to the “Intention-Focused” paradigm for improving bystander CPR performance presented by Panchal *et al*. [36], distal variables such as bystander characteristics based on socio-demographic factors may play a very important role. The example variables presented by the authors may potentially impact bystander beliefs. From this perspective, looking for potential differences in populations originating from administrative areas of different sizes is reasonable because it can affect attitudes, perceived norms, self-efficacy, intentions, perceptions, and barriers and behaviors [36, 37, 38].

However, along the way, the final decision may be influenced by several external factors affecting the decision to start resuscitation; some have already been initially assessed by previous authors [9, 10, 11, 12, 13, 14, 15, 16, 17, 18, 19, 24, 32, 33, 34, 35]. However, it remains difficult to obtain a comprehensive picture of the problem. This carries with it a significant concern about the possibility of generalizing the results of individual studies. Perhaps, although it is not obvious in many areas, we should accept it and adopt a slightly different course of action. The starting point for a better understanding of the problem may be the “Bridge of survival” [24]. The idea of continuous identification and flexibility of actions to increase the chances of survival in out-of-hospital cardiac arrest. In practice, conscious acceptance of the complexity and variability of the research problem concerning the human factor and the factors influencing it. From this perspective, it seems important to search for every variable that, even in the local environment, could impact, or lack thereof, on taking critical actions in saving life in sudden cardiac arrest (SCA). Although this will not always reflect the reality of other populations, the issue is worthy of research efforts in relation to such an important public health problem as cardiac arrest.

## 5. Limitations

The main limitations of this work are its single-center design and the use of self-reported questionnaires for data collection. The sample size is adequate for the number of first-year medical students at the Medical University of Lodz, but the findings may be limited to only this specific population. Attention should also be paid to the fact that administrative regulation laws regarding rural areas in Poland are not based on the number of its inhabitants; consequently, participating in the study, respondents may have inaccurately declared their domicile. Diverse geographical regions and countries may have distinct criteria for classifying residential status or different psychological characteristics of a given population. Future research should include different populations of respondents to avoid generalization errors. Data gathered from self-reporting respondents are also limited due to potential social desirability bias and subjective assessment of reality. Future studies could elaborate on the problem by collecting data with a methodology based on interviews or simulations to reduce the influence of these limitations. Studies with multi-center, cross-cultural, and longitudinal approaches should be carried out to investigate differences in emergency responses across other regions and countries or to observe changes in willingness and barriers following training. Future studies might also employ experimental designs to analyze the causal relationship between psychological barriers and emergency behaviors.

## 6. Conclusions

Overall, respondents did not show a low or significant difference in the declared number or type of barriers, depending on the population size of their home region. The most cited barriers were fear of harming the victim, fear of contracting disease from the victim, and uncertainty about identifying cardiac arrest. Medical students living in cities with over 100,000 inhabitants most frequently reported willingness to perform CPR using chest compressions and rescue breaths for all types of victims. Those who grew up in towns <100,000 residents were less willing to start CPR if the SCA victim would be an unknown adult.

However, these differences may be of low or moderate practical significance. It is likely that studies on other, larger, and more diverse populations, using additional research tools, will be necessary to confirm or exclude the influence of the size of the place of residence on the willingness to perform resuscitation and the related barriers.

## Data Availability

The data presented in this study is available on reasonable request from the corresponding author due to restrictions resulting from the policy of the Bioethics Committee.

## Supplementary Material

Supplementary material associated with this article can be found, in the online version, at https://doi.org/10.31083/j.rcm2512451.
