# Supplementary material for: Barriers and Willingness to Undertake Cardiopulmonary Resuscitation Reported by Medical Students Dependent on Their Place of Residence—A Single-Center Study
Source: Rev Cardiovasc Med. 2024 Dec 23;25(12):451. doi: 10.31083/j.rcm2512451 (PMC11683695; doi:10.31083/j.rcm2512451)
Supplement: Supplementary file 1 [file 2153-8174-25-12-451-s1.zip › RCM26089-Supplementary Material 1.docx]

Barriers to Undertaking Cardiopulmonary Resuscitation

Please fill out the survey. It will take only a few minutes and will contribute to the advancement of science and the improvement of survival rates for victims of sudden cardiac arrest (SCA). This study aims to identify factors influencing fear or reluctance to perform cardiopulmonary resuscitation for a victim with out-of-hospital cardiac arrest, depending on the bystander’s place of residence.

* Required

1. Age (in years) *
2. Gender *

- Male
- Female
- Prefer not to answer

1. Field of study *
2. Province (origin) *
3. Indicate your place of residence size **before starting university studies** *

- Rural area (e.g., countryside village)
- Town with up to 100,000 inhabitants
- City with more than 100,000 inhabitants

1. Have you participated in a first aid course or school training in first aid (e.g., school, driving school, university classes, private, at work)? *

- No, never
- Yes, within the last year
- Yes, more than a year ago

1. To what extent do you think the CPR training you received might be useful in a real-life situation? *

- It will be completely useless
- It will be of little use
- I cannot determine
- It will be sufficiently useful
- It will be very useful

1. Did you practice assessing consciousness and checking for normal breathing during the training (on a manikin or simulator)? *

- Yes
- No

1. Did you practice chest compressions and rescue breaths on an adult manikin during the training? *

- Yes, compressions only
- Yes, compressions and breaths
- No

1. Did you practice chest compressions and rescue breaths on a child manikin during the training? *

- Yes, compressions only
- Yes, compressions and breaths
- No

1. Did you practice using an automated external defibrillator (AED) during the training (using a training AED)? *

- Yes
- No

1. For which of the following individuals would you perform the following actions: assessing consciousness, checking for breathing, and **performing chest compressions** **alone**? (Select all that apply) *

- Adult family member
- Child from the family or close circle
- Stranger child
- Stranger adult
- I would not perform these actions regardless of the circumstances or who the victim is

1. For which of the following individuals would you perform the following actions: assessing consciousness, checking for breathing, and **performing chest compressions and rescue breaths**? (Select all that apply) *

- Adult family member
- Child from the family or close circle
- Stranger child
- Stranger adult
- I would not perform these actions regardless of the circumstances or who the victim is

1. If there is any reason why you think you would not start resuscitation, please specify here (if multiple reasons, separate them with commas): *
2. Would you use an automated external defibrillator (AED) if one were available nearby? *

- Yes
- No

1. Select all statements that define your barriers/fear of starting resuscitation: *

- I lack sufficient knowledge to perform resuscitation.
- I lack sufficient skills to perform resuscitation.
- I lack self-confidence to start resuscitation.
- I fear contracting a disease from the victim.
- I fear contracting coronavirus from the victim.
- I fear that I will panic.
- I fear that I will harm the victim.
- I fear legal consequences of incorrect actions.
- I fear that the victim will be bloodied.
- I fear that the victim will have vomit on their lips.
- I fear that the victim will smell of alcohol.
- I fear that I won't have the physical strength or stamina.
- Low socio-economic status of the victim.
- Advanced age of the victim.
- The victim is a child.
- The victim is a woman.
- Without a specific reason.

1. Check online sources and enter the most accurate number of inhabitants of your place of residence **before starting university studies** (you can use sources such as https://www.polskawliczbach.pl/ or enter the number you know): *
