# Supplementary material for: Barriers and Willingness to Undertake Cardiopulmonary Resuscitation Reported by Medical Students Dependent on Their Place of Residence—A Single-Center Study
Source: Rev Cardiovasc Med. 2024 Dec 23;25(12):451. doi: 10.31083/j.rcm2512451 (PMC11683695; doi:10.31083/j.rcm2512451)
Supplement: Supplementary file 1 [file 2153-8174-25-12-451-s1.zip › RCM26089-Supplementary Material 2.docx]

|  | Beta coefficient | Beta error | -95% CI | +95% CI | Wald test | p-value | Odds ratio | -95% CI | +95% CI |
| --- | --- | --- | --- | --- | --- | --- | --- | --- | --- |
| [Without a specific reason] - The convergence criteria have not been met (too few cases with a value of 1) | | | | | | | | | |
| Intercept | -28.2438 | 1971.30 | -3891.92 | 3835.43 | 0.0002 | 0.9886 | 0 | 0 | NA |
| Age | 0.3992 | 0.1775 | 0.0513 | 0.7472 | 5.0568 | 0.0245 | 1.4907 | 1.0526 | 2.111 |
| Sex [female =1] | -17.6902 | 1650.39 | -3252.4 | 3217.02 | 0.0001 | 0.9914 | 2.08E-8 | 0 | NA |
| Place of residence | 0.5192 | 0.7206 | -0.8932 | 1.9317 | 0.5192 | 0.4712 | 1.6808 | 0.4094 | 6.901 |
| Time since training [>1 year = 1] | 16.4768 | 1971.30 | -3847.19 | 3880.15 | 6.99E-5 | 0.9933 | 14315430 | 0 | NA |
| [Fear of legal consequences] | | | | | | | | | |
| Intercept | 7.2361 | 3.0069 | 1.3428 | 13,1295 | 5.7914 | 0.0161 | 1388.7 | 3.8297 | 503586 |
| Age | -0.4011 | 0.1542 | -0.7033 | -0.0989 | 6.7664 | 0.0093 | 0.6696 | 0.495 | 0.9059 |
| Sex [female =1] | 0.2204 | 0.298 | -0.3637 | 0.8044 | 0.5468 | 0.4596 | 1.2465 | 0.6951 | 2.2354 |
| Place of residence | -0.2732 | 0.1645 | -0.5955 | 0.0492 | 2.7586 | 0.0967 | 0.761 | 0.5513 | 1.0504 |
| Time since training [>1 year = 1] | 0.3436 | 0.2757 | -0.1968 | 0.884 | 1.5527 | 0.2127 | 1.41 | 0.8213 | 2.4207 |
| [Fear of contracting disease from the victim] | | | | | | | | | |
| Intercept | -2.0541 | 1.9185 | -5.8144 | 1.7061 | 1.1463 | 0.2843 | 0.1282 | 0.003 | 5.5076 |
| Age | 0.0904 | 0.0973 | -0.1004 | 0.2812 | 0.8632 | 0.3528 | 1.0947 | 0.9045 | 1.3248 |
| Sex [female =1] | 0.1123 | 0.2702 | -0.4173 | 0.6419 | 0.1726 | 0.6778 | 1.1188 | 0.6588 | 1.9 |
| Place of residence | 0.009 | 0.1512 | -0.2874 | 0.3054 | 0.0035 | 0.9526 | 1.009 | 0.7502 | 1.3572 |
| Time since training [>1 year = 1] | 0.2551 | 0.2543 | -0.2433 | 0.7535 | 1.0061 | 0.3158 | 1.2906 | 0.784 | 2.1244 |
| [Fear of contracting coronavirus] | | | | | | | | | |
| Intercept | -3.9099 | 3.5007 | -10.7711 | 2.9514 | 1.2474 | 0.264 | 0.02 | 2.1E-5 | 19.1318 |
| Age | 0.0924 | 0.1773 | -0.2551 | 0.44 | 0.2718 | 0.6021 | 1.0969 | 0.7749 | 1.5527 |
| Sex [female =1] | -0.1604 | 0.552 | -1.2423 | 0.9214 | 0.0845 | 0.7713 | 0.8518 | 0.2887 | 2.5129 |
| Place of residence | -0.3237 | 0.3208 | -0.9524 | 0.305 | 1.0182 | 0.3129 | 0.7235 | 0.3858 | 1.3567 |
| Time since training [>1 year = 1] | 0.1697 | 0.541 | -0.8906 | 1.23 | 0.0984 | 0.7538 | 1.1849 | 0.4104 | 3.4211 |
| [Fear of vomit] | | | | | | | | | |
| Intercept | 0.6005 | 1.9353 | -3.1926 | 4.3936 | 0.0963 | 0.7563 | 1.823 | 0.0411 | 80.9293 |
| Age | -0.0724 | 0.0983 | -0.2651 | 0.1203 | 0.5421 | 0.4616 | 0.9302 | 0.7671 | 1.1279 |
| Sex [female =1] | 0.3386 | 0.2745 | -0.1993 | 0.8765 | 1.5221 | 0.2173 | 1.403 | 0.8193 | 2.4025 |
| Place of residence | 0.0852 | 0.1527 | -0.2142 | 0.3845 | 0.311 | 0.577 | 1.0889 | 0.8072 | 1.4689 |
| Time since training [>1 year = 1] | 0.3151 | 0.2572 | -0.1891 | 0.8193 | 1.5007 | 0.2206 | 1.3704 | 0.8277 | 2.2689 |
| [Fear of not having enough physical strength] | | | | | | | | | |
| Intercept | -2.5883 | 2.5727 | -7.6306 | 2.4541 | 1.0122 | 0.3144 | 0.0752 | 0.0005 | 11.6358 |
| Age | 0.0624 | 0.1298 | -0.192 | 0.3168 | 0.2312 | 0.6306 | 1.0644 | 0.8253 | 1.3727 |
| Sex [female =1] | 0.6698 | 0.3955 | -0.1053 | 1.445 | 2.8683 | 0.0903 | 1.9539 | 0.9 | 4.2417 |
| Place of residence | -0.2699 | 0.2019 | -0.6656 | 0.1259 | 1.7858 | 0.1814 | 0.7635 | 0.5139 | 1.1342 |
| Time since training [>1 year = 1] | -0.2297 | 0.3322 | -0.8809 | 0.4214 | 0.4782 | 0.4893 | 0.7947 | 0.4144 | 1.5241 |
| [Fear of a victim under the influence of alcohol] | | | | | | | | | |
| Intercept | -3.4045 | 2.2426 | -7.8 | 0.991 | 2.3045 | 0.129 | 0.0332 | 0.0004 | 2.6941 |
| Age | 0.0668 | 0.1124 | -0.1536 | 0.2872 | 0.3533 | 0.5522 | 1.0691 | 0.8577 | 1.3327 |
| Sex [female =1] | 1.0264 | 0.3685 | 0.3041 | 1.7487 | 7.757 | 0.0054 | 2.791 | 1.3554 | 5.7471 |
| Place of residence | -0.1114 | 0.1838 | -0.4717 | 0.2489 | 0.3672 | 0.5445 | 0.8946 | 0.6239 | 1.2826 |
| Time since training [>1 year = 1] | 0.655 | 0.3151 | 0.0374 | 1.2726 | 4.3204 | 0.0377 | 1.9251 | 1.0381 | 3.5701 |
| [Fear of a bloodied victim] | | | | | | | | | |
| Intercept | -6.9484 | 2.3538 | -11.5616 | -2.3351 | 8.7145 | 0.0032 | 0.001 | 9.5245E-6 | 0.0968 |
| Age | 0.2171 | 0.1157 | -0.0096 | 0.4438 | 3.5222 | 0.0606 | 1.2425 | 0.9904 | 1.5586 |
| Sex [female =1] | 0.9261 | 0.4499 | 0.0444 | 1.8078 | 4.2382 | 0.0395 | 2.5247 | 1.0454 | 6.0971 |
| Place of residence | 0.0368 | 0.2214 | -0.3971 | 0.4708 | 0.0277 | 0.8678 | 1.0375 | 0.6723 | 1.6012 |
| Time since training [>1 year = 1] | 0.2705 | 0.3734 | -0.4614 | 1.0025 | 0.5249 | 0.4688 | 1.3107 | 0.6304 | 2.725 |
| [Fear of panicking] | | | | | | | | | |
| Intercept | 1.299 | 2.6775 | -3.9488 | 6.5469 | 0.2354 | 0.6276 | 3.6657 | 0.0193 | 697.0691 |
| Age | -0.1702 | 0.1368 | -0.4383 | 0.0979 | 1.5482 | 0.2134 | 0.8435 | 0.6451 | 1.1029 |
| Sex [female =1] | 1.1047 | 0.3558 | 0.4075 | 1.802 | 9.6435 | 0.0019 | 3.0185 | 1.503 | 6.0618 |
| Place of residence | 0.0397 | 0.1748 | -0.3029 | 0.3822 | 0.0515 | 0.8205 | 1.0405 | 0.7387 | 1.4655 |
| Time since training [>1 year = 1] | 0.2078 | 0.2906 | -0.3617 | 0.7773 | 0.5113 | 0.4746 | 1.2309 | 0.6965 | 2.1756 |
| [Fear of causing harm to the victim] | | | | | | | | | |
| Intercept | -1.3568 | 1.9182 | -5.1164 | 2.4028 | 0.5003 | 0.4794 | 0.2575 | 0.006 | 11.0539 |
| Age | 0.01 | 0.097 | -0.1801 | 0.2002 | 0.0107 | 0.9176 | 1.0101 | 0.8352 | 1.2216 |
| Sex [female =1] | 1.0669 | 0.2821 | 0.514 | 1.6198 | 14.3037 | 0.0002 | 2.9064 | 1.672 | 5.0522 |
| Place of residence | 0.1776 | 0.1559 | -0.128 | 0.4832 | 1.2972 | 0.2547 | 1.1943 | 0.8798 | 1.6213 |
| Time since training [>1 year = 1] | 0.2776 | 0.2625 | -0.237 | 0.7922 | 1.118 | 0.2904 | 1.32 | 0.789 | 2.2082 |
| [Lack of self-confidence] | | | | | | | | | |
| Intercept | 1.0997 | 2.2773 | -3.3638 | 5.5631 | 0.2332 | 0.6292 | 3.0032 | 0.0346 | 260.6345 |
| Age | -0.101 | 0.1158 | -0.328 | 0.1261 | 0.7597 | 0.3834 | 0.904 | 0.7203 | 1.1344 |
| Sex [female =1] | 0.7041 | 0.3034 | 0.1095 | 1.2987 | 5.3864 | 0.0203 | 2.022 | 1.1157 | 3.6644 |
| Place of residence | -0.2626 | 0.1624 | -0.581 | 0.0558 | 2.6139 | 0.1059 | 0.769 | 0.5593 | 1.0573 |
| Time since training [>1 year = 1] | 0.4334 | 0.2734 | -0.1025 | 0.9693 | 2.5125 | 0.1129 | 1.5425 | 0.9026 | 2.6362 |
| [Lack of adequate knowledge] | | | | | | | | | |
| Intercept | 3.2615 | 2.4496 | -1.5397 | 8.0626 | 1.7727 | 0.183 | 26.0879 | 0.2145 | 3173.55 |
| Age | -0.1865 | 0.1249 | -0.4314 | 0.0584 | 2.2282 | 0.1355 | 0.8299 | 0.6496 | 1.0601 |
| Sex [female =1] | -0.0423 | 0.2901 | -0.6107 | 0.5262 | 0.0212 | 0.8842 | 0.9586 | 0.5429 | 1.6925 |
| Place of residence | -0.1571 | 0.1619 | -0.4745 | 0.1602 | 0.9417 | 0.3318 | 0.8546 | 0.6222 | 1.1738 |
| Time since training [>1 year = 1] | -0.0831 | 0.2705 | -0.6133 | 0.4471 | 0.0943 | 0.7587 | 0.9203 | 0.5416 | 1.5638 |
| [Uncertainty if cardiac arrest was correctly identified] | | | | | | | | | |
| Intercept | -0.0671 | 1.8907 | -3.7729 | 3.6387 | 0.0013 | 0.9717 | 0.9351 | 0.023 | 38.0415 |
| Age | -0.0375 | 0.0959 | -0.2254 | 0.1505 | 0.1526 | 0.6961 | 0.9632 | 0.7982 | 1.1624 |
| Sex [female =1] | 0.5276 | 0.274 | -0.0094 | 1.0646 | 3.7087 | 0.0541 | 1.6949 | 0.9907 | 2.8996 |
| Place of residence | 0.0859 | 0.1525 | -0.2129 | 0.3848 | 0.3177 | 0.573 | 1.0897 | 0.8082 | 1.4693 |
| Time since training [>1 year = 1] | 0.4166 | 0.2571 | -0.0874 | 0.9205 | 2.6245 | 0.1052 | 1.5167 | 0.9163 | 2.5107 |
| [Low socio-economic status of the victim] | | | | | | | | | |
| Intercept | 0.2864 | 3.5012 | -6.5758 | 7.1486 | 0.0067 | 0.9348 | 1.3316 | 0.0014 | 1272.28 |
| Age | -0.1389 | 0.179 | -0.4897 | 0.2119 | 0.6024 | 0.4377 | 0.8703 | 0.6128 | 1.236 |
| Sex [female =1] | 0.1041 | 0.4258 | -0.7305 | 0.9387 | 0.0597 | 0.8069 | 1.1097 | 0.4817 | 2.5565 |
| Place of residence | -0.2907 | 0.2392 | -0.7595 | 0.1782 | 1.4765 | 0.2243 | 0.7478 | 0.4679 | 1.195 |
| Time since training [>1 year = 1] | 1.349 | 0.4757 | 0.4167 | 2.2814 | 8.043 | 0.0046 | 3.8537 | 1.517 | 9.79 |
| [Advanced age of the victim] | | | | | | | | | |
| Intercept | -5.1846 | 3.9156 | -12.8589 | 2.4898 | 1.7532 | 0.1855 | 0.0056 | 2.60E-6 | 12.0588 |
| Age | 0.0682 | 0.1964 | -0.3168 | 0.4531 | 0.1204 | 0.7286 | 1.0705 | 0.7285 | 1.5732 |
| Sex [female =1] | 0.1529 | 0.6562 | -1.1332 | 1.439 | 0.0543 | 0.8158 | 1.1652 | 0.322 | 4.2164 |
| Place of residence | -0.1243 | 0.3719 | -0.8532 | 0.6046 | 0.1118 | 0.7382 | 0.8831 | 0.426 | 1.8305 |
| Time since training [>1 year = 1] | 1.3392 | 0.7914 | -0.212 | 2.8904 | 2.8632 | 0.0906 | 3.816 | 0.809 | 18.0005 |
| [The victim is a child] | | | | | | | | | |
| Intercept | -4.2454 | 3.1991 | -10.5155 | 2.0247 | 1.7611 | 0.1845 | 0.0143 | 2.71E-5 | 7.5735 |
| Age | 0.0263 | 0.16 | -0.2873 | 0.3399 | 0.027 | 0.8695 | 1.0266 | 0.7503 | 1.4047 |
| Sex [female =1] | 1.2737 | 0.5737 | 0.1493 | 2.3981 | 4.9289 | 0.0264 | 3.574 | 1.161 | 11.0025 |
| Place of residence | 0.1711 | 0.2544 | -0.3275 | 0.6698 | 0.4526 | 0.5011 | 1.1867 | 0.7207 | 1.9538 |
| Time since training [>1 year = 1] | 0.4392 | 0.4264 | -0.3967 | 1.275 | 1.0605 | 0.3031 | 1.5514 | 0.6726 | 3.5787 |
| [The victim is a woman] | | | | | | | | | |
| Intercept | -10.5554 | 3.8007 | -18.0047 | -3.1061 | 7.7128 | 0.0055 | 2.60E-5 | 1.52E-8 | 0.0448 |
| Age | 0.4623 | 0.203 | 0.0644 | 0.8601 | 5.1869 | 0.0228 | 1.5877 | 1.0666 | 2.3635 |
| Sex [female =1] | -1.1348 | 0.9229 | -2.9436 | 0.674 | 1.5121 | 0.2188 | 0.3215 | 0.0527 | 1.962 |
| Place of residence | -1.0814 | 0.6616 | -2.3781 | 0.2153 | 2.6716 | 0.1022 | 0.3391 | 0.0927 | 1.2403 |
| Time since training [>1 year = 1] | 0.0934 | 0.941 | -1.751 | 1.9377 | 0.0098 | 0.921 | 1.0979 | 0.1736 | 6.9427 |
| [There are many witnesses at the scene] | | | | | | | | | |
| Intercept | -1.5846 | 3.496 | -8.4366 | 5.2674 | 0.2054 | 0.6504 | 0.205 | 0.0002 | 193.9125 |
| Age | -0.0346 | 0.1777 | -0.383 | 0.3137 | 0.038 | 0.8455 | 0.966 | 0.6818 | 1.3685 |
| Sex [female =1] | -0.1733 | 0.471 | -1.0964 | 0.7498 | 0.1354 | 0.7129 | 0.8409 | 0.3341 | 2.1165 |
| Place of residence | 0.1231 | 0.269 | -0.4042 | 0.6504 | 0.2094 | 0.6472 | 1.131 | 0.6675 | 1.9164 |
| Time since training [>1 year = 1] | -0.4481 | 0.4481 | -1.3264 | 0.4302 | 1.0001 | 0.3173 | 0.6388 | 0.2654 | 1.5375 |
